# Exploring Immune-Related Prognostic Signatures in the Tumor Microenvironment of Colon Cancer

**DOI:** 10.3389/fgene.2022.801484

**Published:** 2022-02-24

**Authors:** Lichao Cao, Tong Li, Ying Ba, Erfei Chen, Jin Yang, Hezi Zhang

**Affiliations:** ^1^ Provincial Key Laboratory of Biotechnology of Shaanxi Province, Northwest University, Xi’an, China; ^2^ Key Laboratory of Resource Biology and Biotechnology in Western China, Ministry of Education, School of Life Sciences, Northwest University, Xi’an, China; ^3^ Shenzhen Nucleus Gene Technology Co., Ltd., Shenzhen, China

**Keywords:** colon cancer, tumor immune microenvironment, prognostic model, immunotherapy, TCGA-COAD, GEO

## Abstract

**Background:** Colon cancer is a common malignant tumor with poor prognosis. The aim of this study is to explore the immune-related prognostic signatures and the tumor immune microenvironment of colon cancer.

**Methods:** The mRNA expression data of TCGA-COAD from the UCSC Xena platform and the list of immune-related genes (IRGs) from the ImmPort database were used to identify immune-related differentially expressed genes (DEGs). Then, we constructed an immune-related risk score prognostic model and validated its predictive performance in the test dataset, the whole dataset, and two independent GEO datasets. In addition, we explored the differences in tumor-infiltrating immune cell types, tumor mutation burden (TMB), microsatellite status, and expression levels of immune checkpoints and their ligands between the high-risk and low-risk score groups. Moreover, the potential value of the identified immune-related signature with respect to immunotherapy was investigated based on an immunotherapeutic cohort (Imvigor210) treated with an anti-PD-L1 agent.

**Results:** Seven immune-related DEGs were identified as prognostic signatures. The areas under the curves (AUCs) of the constructed risk score model for overall survival (OS) were calculated (training dataset: 0.780 at 3 years, 0.801 at 4 years, and 0.766 at 5 years; test dataset: 0.642 at 3 years, 0.647 at 4 years, and 0.629 at 5 years; and the whole dataset: 0.642 at 3 years, 0.647 at 4 years, and 0.629 at 5 years). In the high-risk score group of the whole dataset, patients had worse OS, higher TMN stages, advanced pathological stages, and a higher *TP53* mutation rate (*p* < 0.05). In addition, a high level of resting NK cells or M0 macrophages, and high TMB were significantly related to poor OS (*p* < 0.05). Also, we observed that high-risk score patients had a high expression level of *PD-L1*, *PD-1*, and *CTLA-4* (*p* < 0.05). The patients with high-risk scores demonstrated worse prognosis than those with low-risk scores in multiple datasets (GSE39582: *p* = 0.0023; GSE17536: *p* = 0.0008; immunotherapeutic cohort without platinum treatment: *p* = 0.0014; immunotherapeutic cohort with platinum treatment: *p* = 0.0027).

**Conclusion:** We developed a robust immune-related prognostic signature that performed great in multiple cohorts and explored the characteristics of the tumor immune microenvironment of colon cancer patients, which may give suggestions for the prognosis and immunotherapy in the future.

## Introduction

Colon cancer is known as one of the most malignant tumors with a high mortality rate worldwide (Siegel et al., 2018). Despite the recent progress in diagnosis and therapy, the overall prognosis for colon cancer patients remains poor because effective biomarkers for prognosis prediction are lacking (Keum and Giovannucci, 2019). Therefore, it is urgent and essential to explore valuable prognostic signatures and therapeutic targets for colon cancer.

Immunotherapy takes advantage of the body’s own immune system to attack cancer, which has become a powerful and promising clinical strategy for treating various tumors (Riley et al., 2019), including colon cancer(Chalabi et al., 2020; Lichtenstern et al., 2020; Ghonim et al., 2021). Immune checkpoint inhibitors (ICIs), a typical type of immunotherapy, function through inhibiting negative regulatory receptors, such as programmed cell death 1 (*PD-1*) and cytotoxic T lymphocyte antigen 4 (*CTLA4*), and thereby activates antitumor immunity (Tolba, 2020). However, only a fraction of patients were benefited from immunotherapy due to the heterogeneity and complexity of the tumor immune microenvironment (Dienstmann et al., 2017; Wang et al., 2019). Although it has been proved that IRGs were associated with the development of colon cancer (Cereda et al., 2016; Yu et al., 2019), these insights have not been applied to clinical practice. Recently, using bioinformatics and machine learning methods, various types of immune-related biomarkers have been found to be associated with the prognosis of colon cancer, such as long non-coding RNAs (Yilin Lin et al., 2020), cell infiltration (Zhou et al., 2019), and IRGs (Chen et al., 2020). However, the molecular characteristics describing the tumor immune microenvironment need to be further investigated due to their potential of prognosis and immunotherapy of colon cancer.

In this study, we constructed and validated a robust immune-related prognostic model based on TCGA-COAD cohorts and two independent GEO datasets. Additionally, we explored the relationship between the constructed prognostic model and colon cancer patients’ clinical and pathological features. In addition, we analyzed the characteristics of the tumor immune microenvironment, including tumor-infiltrating cell composition, TMB, *TP53* mutation rates, and the mRNA expression levels of *PD-1/PD-L1/CLTA4*. Furthermore, the immune-related signature was also significantly associated with OS in patients with anti-PD-L1 treatment, and colon cancer patients with low-risk scores may be more sensitive to ICI therapy. These findings may provide new insights toward novel therapeutic targets for colon cancer.

## Materials and Methods

### Data Acquiring

#### TCGA Cohorts and the List of Immune-Related Genes

The mRNA sequencing data, mutation profiling data, and clinical information were downloaded from the UCSC Xena platform (https://xenabrowser.net/datapages/). Subsequently, the samples (n = 471) were divided into normal (*n* = 39) and tumor groups (*n* = 432), and the detailed information is shown in Supplementary Table S1. The list of immune-related genes was acquired from the ImmPort database (https://immport.niaid.nih.gov/), with a total of 1509 genes.

#### GEO Cohort for External Validation

Two independent datasets (GSE39582 and GSE17536) were downloaded from the GEO database (https://www.ncbi.nlm.nih.gov/geo/). The GSE39582 included 556 colon cancer samples, and GSE17536 included 177 colon cancer samples, with clinical and survival information. The detailed information is shown in Supplementary Tables S2, S3, respectively.

#### Immunotherapeutic Cohort

An immunotherapeutic cohort (IMvigor210) was obtained from a published study (Mariathasan et al., 2018), which investigated the clinical activity of the PD-L1 blockade with atezolizumab (anti-PD-L1 McAb) in urothelial cancer. The detailed clinical information and gene expression profile of the cohort were available according to the guideline on http://research-pub.gene.com/IMvigor210CoreBiologies using the IMvigor210CoreBiologies R package. We divided the samples into platinum-treated (*N* = 105) and non-platinum-treated datasets (*N* = 237) according to whether they received platinum-based chemotherapy or not, and the detailed information is shown in Supplementary Table S4.

### Screening Immune-Related DEGs

DEGs between normal and tumor groups were screened using the *limma* R package (Ritchie et al., 2015), with the cutoff criteria set as | log2 fold change (FC)| >0.585 and adjusted *p-value* < 0.05. The immune-related DEGs were obtained by overlapping the IRGs and DEGs. In order to investigate biological pathways correlated with immune response, we performed gene ontoloy (GO) functional annotations and Kyoto Encyclopedia of Genes and Genomes (KEGG) enrichment analysis on immune-related DEGs using the *clusterProfiler* R package (Yu et al., 2012).

### Construction and Validation of the Immune-Related Prognostic Model for Colon Cancer

The whole dataset (*n* = 432) with all tumor samples was randomly divided into training dataset (*n* = 216) and test dataset (*n* = 216) with a 1:1 ratio. As shown in the Supplementary Table S5, there was no significant difference among the whole dataset, the training dataset, and test dataset for most clinical-pathological factors. The training dataset was used to identify the prognostic signature and constructed a prognostic risk model. First, we identified the candidate prognostic signature using the univariable Cox proportional hazards regression model and *Survival* R package. To avoid over-fitting, all genes with *p-value* < 0.05 were involved in the subsequent least absolute shrinkage and selection operator (LASSO) analysis using the *glmnet* R package. The association between the mRNA expression level of the filtered candidate prognostic genes and patients’ OS was further investigated using Kaplan–Meier analysis. Then, the multivariate Cox regression analysis was conducted to determine each independent prognostic indicator. Accordingly, the coefficient of the immune-related indicator was obtained from the multivariate Cox results. A formula for the immune-related risk score model was established to predict patient survival:

risk score=ΣCox coefficient of gene χi×scale expression value of gene χi.

To evaluate the predictive efficiency of the constructed immune-related risk score model, we plotted a receiver operating characteristic (ROC) curve to quantify the area under the curve (AUC) using the *survivalROC* R package. Also, we selected the turning point of the ROC curve with the most significant difference between true positive and false positive as the optimal cutoff risk score. Patients above the cutoff value belong to the high-risk group, while patients below it belong to the low-risk group. In addition, Kaplan–Meier curves were plotted to distinguish the two groups using the *survminer* R package.

Moreover, the test dataset and the whole dataset were used to validate the prognostic capability of the immune-related signature. Similarly, the two datasets were divided into high- and low-risk groups based on the constructed risk score model. Next, the ROC and Kaplan–Meier curves were plotted to validate the predictive accuracy of the risk score model. Then, the nomogram was constructed using the whole dataset based on the risk score model and clinical factors, including the age, gender, microsatellite status, and tumor stage. The constructed nomogram was further assessed by calibration. Additionally, the associations between the immune-related constructed risk score model and the clinical and pathological characteristics, including advanced pathological stages and TNM stages, were explored by using the Wilcoxon test. Additionally, the constructed model was further validated using GEO datasets with accession numbers GSE39582 and GSE17536.

### Estimation and Comparison of Tumor-Infiltrating Immune Cell Type Fractions

The whole dataset was divided into high- and low-risk groups according to the constructed risk model, and the CIBERSORT algorithm was conducted to access the proportions of 22 types of tumor-infiltrating immune cells using the normalized gene expression matrixes and running with 1000 permutations (Newman et al., 2015). Subsequently, the comparison of immune landscape between the high- and low-risk groups was evaluated using the unpaired *t*-test. The significant differential immune cell types (*p-value* < 0.001) were further assessed for their relationship with OS using Kaplan–Meier curves.

### Characteristics of Immunotherapy-Related Predictors for Colon Cancer Patients

We first calculated the TMB value and visualized the mutation profiles of the high- and low-risk groups of the whole dataset using the *maftools* R package (Mayakonda et al., 2018). The unpaired *t*-test statistically analyzed the differences of the TMB between the high- and low-risk groups. In addition, the OS between the high- and low-risk groups was calculated using the Kaplan–Meier method. Moreover, the Wilcoxon test was used to compare the mRNA levels of immune checkpoints and their ligands between the high- and low-risk groups.

### Exploring the Associations Between the Microsatellite Status and the Constructed Prognostic Model

The whole dataset, after removing the samples without microsatellite status information, was used for further analysis based on the constructed prognostic model. Subsequently, the samples were divided into MSI-H and MSS/MSI-L groups according to the microsatellite status information extracted from the phenotypic data, and the Wilcoxon test was performed to analyze the difference of the level of risk score between the MSI-H and MSS/MSI-L groups. Moreover, the OS between the MSI-H group and MSI-L/MSS group was calculated using the Kaplan–Meier method.

### The Role of the Immune-Related Prognostic Signature in Immunotherapy

In order to investigate the potential value of the identified immune-related signature with respect to immunotherapy, we obtained the gene expression profiles and corresponding clinical features from an immunotherapeutic cohort (Imvigor210) treated with anti-PD-L1 agent, which was divided into platinum-treated and non-platinum-treated datasets. We first validated the constructed immune-related prognostic model using the platinum-treated and non-treated datasets, respectively. Then, the complete response (CR) or partial response (PR) patients were categorized as responders and compared with non-responders, who displayed stable (SD) or progressive disease (PD), and the risk score of each patient was calculated based on the constructed risk score model. Subsequently, we statistically analyzed the distribution of the risk score between the responders and non-responders. Moreover, we further compared the tumor mutation load and neoantigen burden between high-and low-risk groups using the Wilcoxon test.

## Results

### Identification of Immune-Related DEGs

**FIGURE 1:**
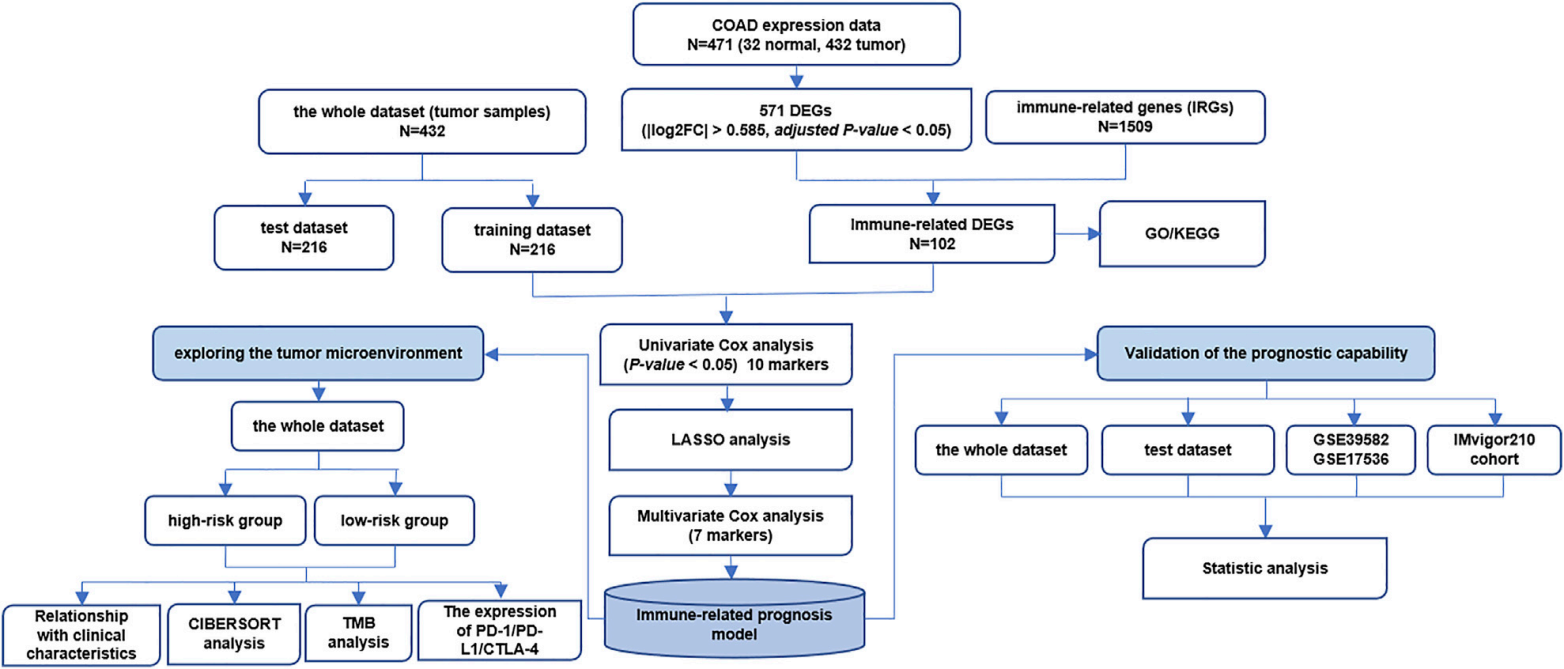
The whole flow chart of data analysis.

**FIGURE 2:**
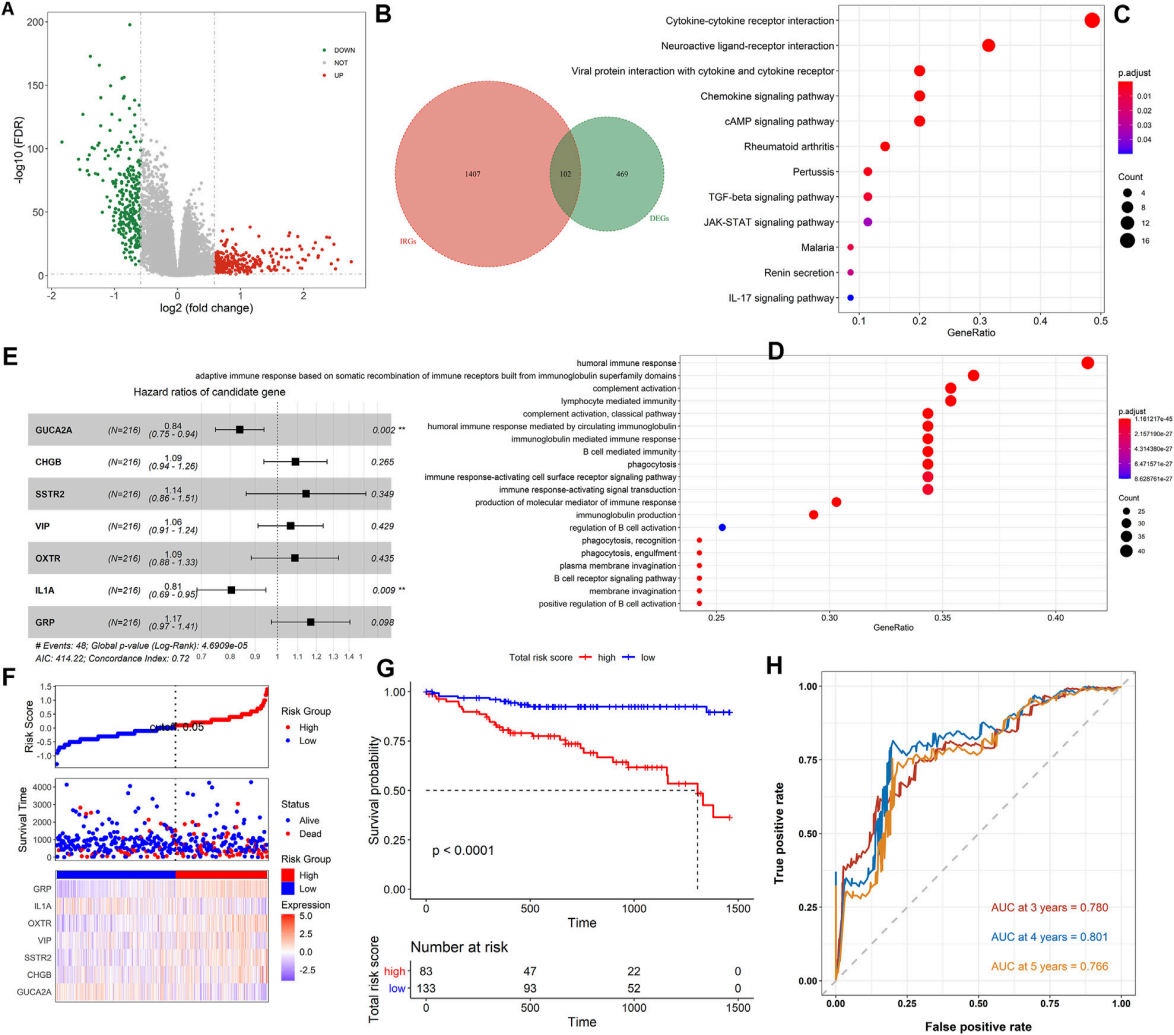
Analysis of immune-related DEGs and construction of the immune-related prognostic model. **(A)** The volcano plot on all DEGs between the tumor and normal samples. The green dots represent downregulated genes, while the red dots represent upregulated genes. **(B)** The Venn diagram of the intersection between the DEGs and IRGs. **(C)** The KEGG pathway enrichment analysis of immune-related DEGs. **(D)** GO analysis of immune-related DEGs. **(E)** The forest plot showed the results of multivariate Cox analysis. **(F)** The distribution of the high- and low-risk score groups and their relationship with OS, and the expression pattern of seven prognostic signatures in high- and low-risk score groups. **(G)** The Kaplan–Meier curve revealed that OS in the low-risk score group was significantly higher than that in the high-risk score group. **(H)** Time-dependent ROC curve analysis of the immune-related risk score model.

A flow chart of the whole analysis pipeline is shown in Figure 1. A total of 571 DEGs (275 upregulated and 296 downregulated) were screened by comparing between tumor and normal groups (Figure 2A). After the intersection with 1509 IRGs, 102 immune-related DEGs were obtained (Figure 2B), of which 83 genes were downregulated, and 19 genes were upregulated. Detailed information is shown in Supplementary Table S6. Subsequently, functional and pathway enrichment analyses were performed using the *clusterProfiler* R package. KEGG analysis results indicated that the immune-related DEGs were significantly enriched in terms associated with the cytokine–cytokine receptor interaction, neuroactive ligand–receptor interaction, and IL-17 signaling pathway (Figure 2C), while GO related to humoral immune response was mediated by circulating immunoglobulin, humoral immune response, and immunoglobulin-mediated immune response (Figure 2D).

### Construction of the Immune-Related Risk Score Model and the Evaluation of its Prognostic Ability

To explore the prognostic value of the immune-related DEGs, we performed the univariate Cox regression analysis. A total of 10 genes were significantly related to OS status, and 7 genes with the maximum prognostic value were further identified using LASSO regression analysis (Supplementary Figures S1A,B). The mRNA expression level of the seven genes was significantly associated with patients’ OS (*GUCA2A: p* = 0.013; *CHGB: p* = 0.05; *SSTR2: p* = 0.017; *VIP: p* = 0.0074; *OXTR: p* = 0.001; *IL1A: p* = 0.0035; and *GRP: p* = 0.016), and the higher expression level of *IL1A* and *GUCA2A* was associated with a better patients’ OS, while the other five genes were opposite (Supplementary Figure S1C–I)*.* Then, we conducted the multivariate Cox regression analysis and established an immune-related risk score model based on the training dataset, and the hazard ratio of each gene is shown in Figure 2E. The colon cancer patients were divided into high- and low-risk groups according to the risk score calculated using the formula described in Materials and Methods. The scatter diagram in Figure 2F revealed that the OS tended to become worse with the increase of risk score, and the proportion of death in the high-risk group (the proportion of red dot and blue dot on the right side) was higher than that in the low-risk group. The heatmap in Figure 2F showed that the expression of *IL1A* and *GUCA2A* was low in the low-risk group and high in the high-risk group, while the trend of the other five genes was opposite. The Kaplan–Meier analysis results showed that high-risk score patients had worse OS than low score patients (*p* < 0.0001, Figure 2G). The prognostic accuracy of the risk score model was investigated as a continuous variable (Figure 2H). The AUC of the prognostic model for OS was 0.780 at 3 years, 0.801 at 4 years, and 0.766 at 5 years, indicating its excellent prediction performance.

### Validation and Assessment of the Immune-Related Prognostic Signatures

**FIGURE 3:**
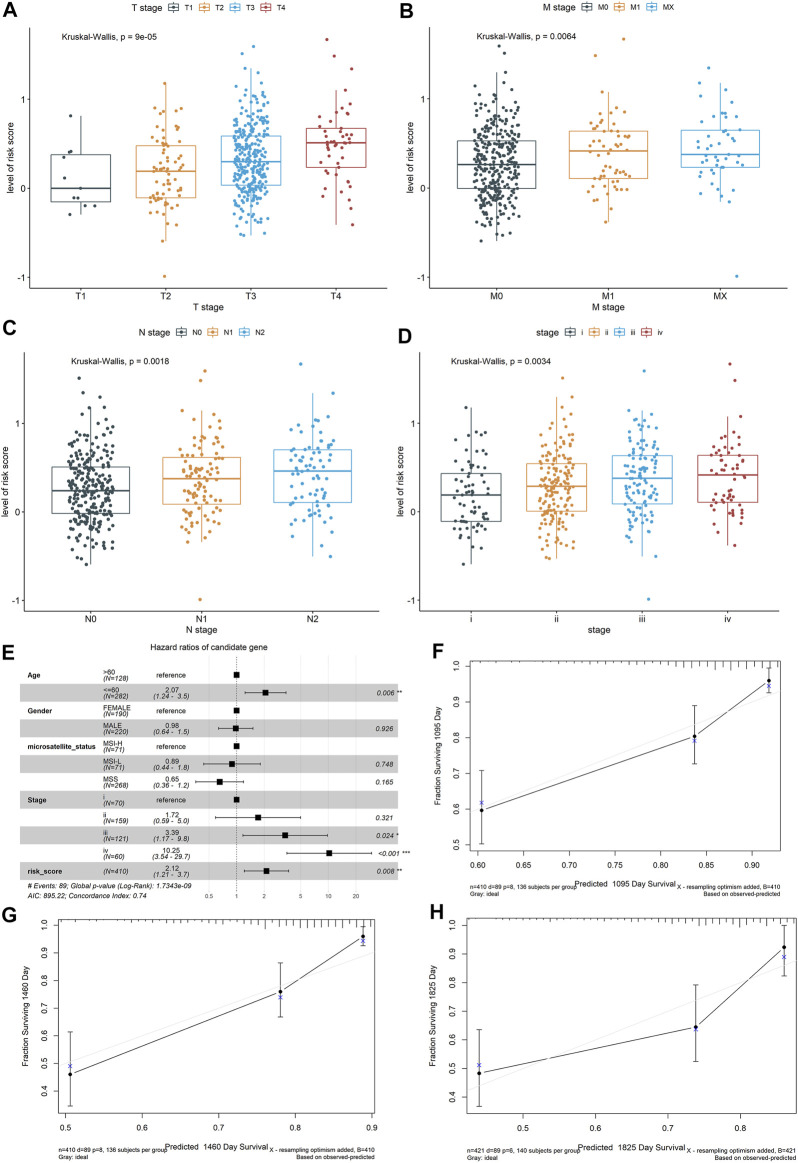
Exploring the relationship between the risk score of the colon cancer patients and clinical and pathological characteristics, including **(A)** T stages, **(B)** M stages, **(C)** N stages, and **(D)** advanced pathological stages, based on the whole dataset. Construction and validation of a nomogram. **(E)** Forest plots showed the associations between patients’ characteristics and OS. **(F–H)** The calibration plot of the nomogram to predict the probability of OS at 3, 4, and 5 years.

To determine if the constructed risk core model is consistent in different populations, we performed an identical formula using the test dataset and the whole dataset. Consistent with the findings in the training dataset, patients categorized into the high-risk score group had worse OS than the patients in the low-risk score group (*p* < 0.05, Supplementary Figures S2A,C). The areas under the curves (AUCs) of the prognostic model were 0.642 for 3-year OS, 0.647 for 4-year OS, and 0.629 for 5-year OS using the test dataset, and 0.626 for 3-year OS, 0.663 for 4-year OS, and 0.661 for 5-year OS using the whole dataset (Supplementary Figures S2B,D). The Wilcoxon test showed that the higher risk score was associated with a higher T stage (*p* = 0.00009), N stages (*p* = 0.0018), metastasis (*p* = 0.0064), and advanced pathological stage (*p* = 0.0034) based on the whole dataset (Figures 3A–D).

To further validate the robustness of the prognostic signatures and improve the accuracy of the performance of the risk score model, we constructed a nomogram that integrated the immune-related risk score and clinical information, including the age, sex, microsatellite status, and tumor stage to quantitatively predict the prognosis of colon cancer patients in the whole dataset. In the nomogram, the score for each variable can be found on the point scale, so that it is easy to estimate the probability of survival at 3, 4, and 5 years by calculating the total score (Supplementary Figure S2E). The forest plot showed that patient’s characters, including the age (>60), tumor stage (III and IV), and risk score were significantly associated with the OS (*p-value* < 0.05, Figure 3E). The calibration curves revealed that the predictive curves were close to the ideal curve (Figures 3F–H), indicating good performance. Furthermore, the predictive accuracy of this nomogram (C-index: 0.74) was higher than that of the risk score model (C-index: 0.72).

### Exploring the Tumor Immune Microenvironment in Colon Cancer Patients

**FIGURE 4:**
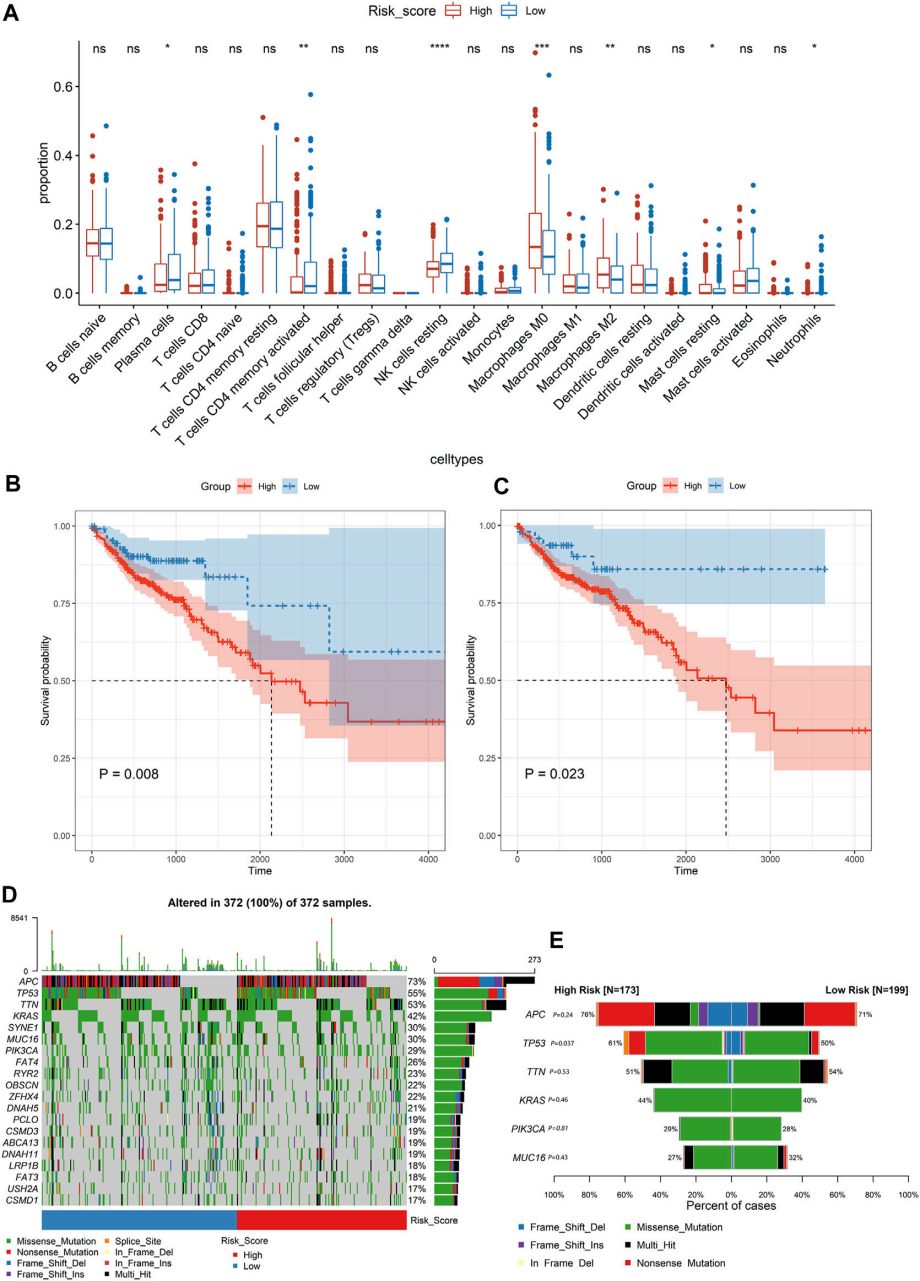
Analysing the immune cell types and mutation profiles in high- and low-risk groups based on the whole dataset. **(A)** Comparing the difference of the proportions of immune cells between the low-risk group and high-risk group using the Wilcoxon test. The values of *P* were labeled above each boxplot with asterisks (*p < 0.05, **p < 0.01, ***p < 0.001, ****p < 0.0001). **(B, C)** The Kaplan–Meier analysis of the relationship between the level of resting NK cells and M0 macrophages with patients’ OS. **(D)** The mutation profiles of colon cancer patients in high- and low-risk groups. **(E)** Comparison of the mutation rate between the high-risk group and low-risk group.

Based on the CIBERSORT algorithm, we estimated the proportions of 22 types of immune cells in each colon cancer patient. Then, we compared the proportions of immune cells between the low-risk group and high-risk group, and the significant differences were found in resting NK cells, M0 macrophages, M2 macrophages, CD4 memory-activated T cells, plasma cells, resting mast cells, and neutrophils. Among them, the resting NK cells and M0 macrophages were the most significant, with *p* < 0.001 (Figure 4A). The Kaplan–Meier curve revealed that a high level of resting NK cells or M0 macrophages was significantly related to poor OS (*p* < 0.05, Figures 4B,C).

**FIGURE 5:**
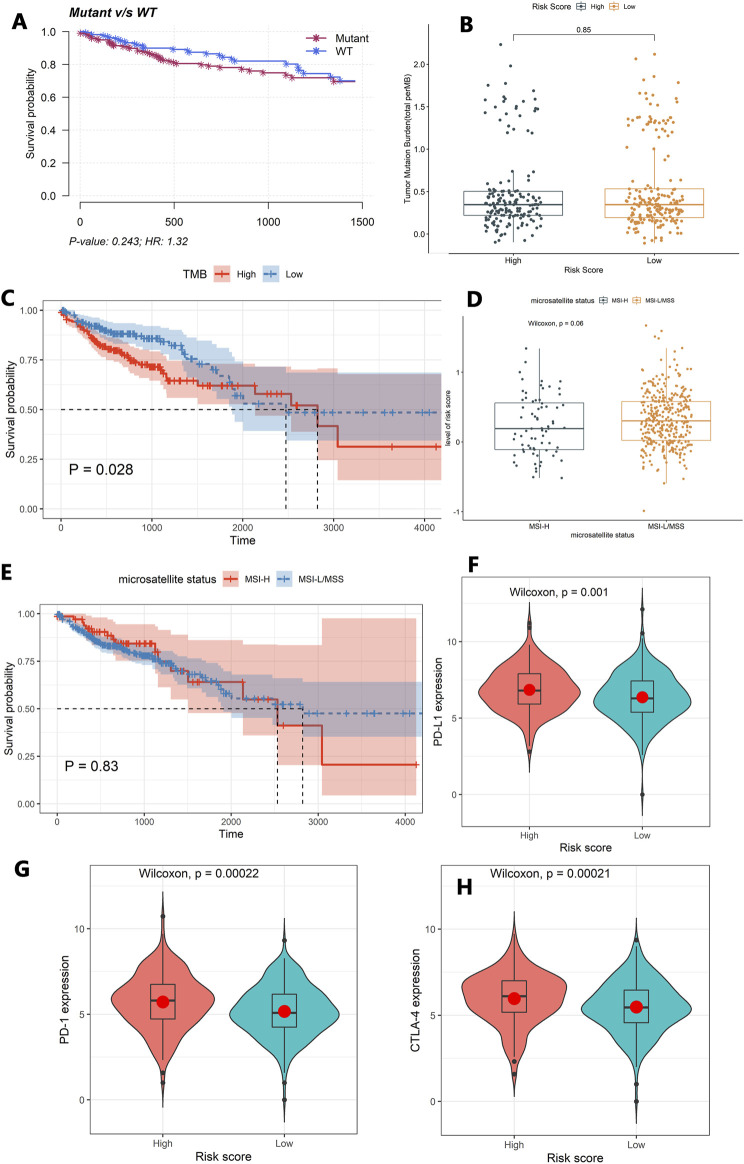
Exploring the tumor immune microenvironment in colon cancer patients. **(A)** The association between the *TP53* status and patients’ OS. **(B)** The difference of TMB between the high-risk group and the low-risk group. **(C)** The association between TMB and patients’ OS. **(D)** The difference in the level of the risk score between the MSI-H and MSI-L/MSS groups. **(E)** The association between the microsatellite status and patients’ OS. **(F–H)** Comparison of the expression levels of the immune checkpoints and their ligands between the high-risk score group and low-risk score group. **(F)** The expression of PD-L1, **(G)** the expression of PD-1, and **(H)** the expression of CTLA-4.

The mutation profiles of each colon cancer patient were plotted using the whole dataset. As shown in Figure 4D, the top 20 significantly mutated genes were *APC, TP53, TTN, KRAS, SYNE1, MUC16, PIK3CA, FAT4, RYR2, OBSCN, ZFHX4, DNAH5, PCLO, CSMD3, ABCA13, DNAH11, LRP1B, FAT3, USH2A*, and *CSMD1*. Among them, the mutation rate of *TP53* was significantly different between the high-risk score group and low-risk group (*p* = 0.037, Figure 4E). However, the *TP53* status was not significantly related to patients’ OS (Figure 5A). Besides, we calculated the TMB of each sample and found that there was no significant difference between the high-risk group and the low-risk group (*p* = 0.85, Figure 5B). However, we observed that high TMB was significantly related to poor patients’ OS (Figure 5C). Additionally, the Wilcoxon test statistically analyzed the difference in the level of risk scores between the MSI-H and MSI-L/MSS groups, and the result showed the difference was not significant (*p* = 0.06, Figure 5D). As shown in Figure 5E, the microsatellite status cannot be used as an independent prognostic factor (*p* = 0.83).

Moreover, the Wilcoxon test was used to compare the expression levels of the immune checkpoints and their ligands between the high-risk score group and low-risk score group. The high-risk score group had a high expression level of *PD-L1* (*p* = 0.001), *PD-1*(*p* = 0.00022), and *CTLA-4* (*p* = 0.00021, Figures 5F–H).

### Validation of the Prognostic Signature Using GEO Datasets

In order to investigate the applicability in multiple cohorts based on different platforms, we further verified the constructed risk score model using two independent external GEO datasets. We found that patients with high-risk scores demonstrated worse prognosis than those with low-risk scores (GSE39582: *p* = 0.0023, Supplementary Figure S3; GSE17536: *p* = 0.0008, Supplementary Figure S4). The AUCs of the prognostic model were 0.577 for 3-year OS, 0.569 for 4-year OS, and 0.568 for 5-year OS using the GSE39582 dataset (Supplementary Figure S3A), and 0.578 for 3-year OS, 0.699 for 4-year OS, and 0.657 for 5-year OS using GSE17536 (Supplementary Figure S4A). The Wilcoxon test showed that a higher risk score was associated with a higher T stage (*p* = 0.0041), metastasis (*p* = 0.04), N stages (*p* = 0.037), and advanced pathological stages (*p* = 0.033) using GSE39582 (Supplementary Figures S3C–F). It was also found that a higher risk score was associated with a higher advanced pathological stage (*p* = 0.029) in GSE17536 (Supplementary Figure S4C), without obtaining the TMN stage data.

### The Prognostic Signature in the Role of ICI Treatment

In the immunotherapeutic cohort, patients with a low-risk score exhibited a significantly prolonged survival rate (non-platinum-treated dataset: *p* = 0.0014, Supplementary Figure S5A; platinum-treated dataset: *p* = 0.033, Supplementary Figure S6A). Patients without platinum treatment indicated marked clinical benefits from immunotherapy in the low-risk score group compared to those with a high-risk score (*p* = 0.0027, Supplementary Figure S5B), but not significantly in patients with platinum treatment (*p* = 0.44, Supplementary Figure S6B). Further analysis revealed that a higher tumor mutation load in patients with platinum treatment was significantly associated with a low-risk score (*p* = 0.039, Supplementary Figure S6C), but not in patients without platinum treatment (*p* = 0.21, Supplementary Figure S5C). Interestingly, higher neoantigen burden in patients without platinum treatment was significantly associated with a low-risk score (*p* = 0.025, Supplementary Figure S5D), but not in patients with platinum treatment (*p* = 0.5, Supplementary Figure S6D).

## Discussion

The immune cells within the tumor microenvironment (TME) function play a key role in tumorigenesis (Lei et al., 2020). Growing evidence has revealed the therapeutic potential of ICIs in colon cancer (Kimura et al., 2020; Wang et al., 2020). However, the limited knowledge on the characteristics of the TME, to some extent, hindered the development of the application of immunotherapy. In recent years, many efforts have been made to identify immune-related biomarkers for the diagnosis and prognosis of colon cancer (Zhou et al., 2019; Laghi et al., 2020; Li et al., 2020). However, more reliable biomarkers still need to be explored to maximize the application of immunotherapy.

In this study, we developed a prognostic risk score model based on seven IRGs, named *GUCA2A, CHGB, SSTR2, VIP, OXTR, IL1A,* and *GRP*, which has been verified in multiple cohorts across different platforms. Among them, *GUCA2A, VIP,* and *OXTR* have been demonstrated to be significantly associated with the prognosis of colon cancer (Zhang et al., 2019; Houxi Xu et al., 2020; Kang Lin et al., 2020; Zhang et al., 2020). A previous study reported that guanylyl cyclase C (*GUCY2C*) could act as a tumor suppressor and play an important role in orchestrating intestinal homeostatic mechanisms, which could be used as a therapeutic target for colon cancer patients (such as the FDA-approved oral *GUCY2C* ligand linaclotide, Linzess™) (Pattison et al., 2016). *GUCA2A* may be considered as a potential marker for the prognosis and therapeutic target in colon cancer by binding and activating *GUCY2C.* As a precursor of regulatory peptide, the relationship between *CHGB* and tumor is not clear. However, *CHGB* was suggested to be an immune-related signature for low-grade glioma (Liu et al., 2021) and head and neck squamous cell carcinoma (Zhang et al., 2021). Previous studies also experimentally demonstrated that an abnormal expression of *CHGB* was associated with aggressive VHL-associated pancreatic neuroendocrine tumors (validated by immunohistochemistry) (Weisbrod et al., 2013), pancreatic cancer (validated by qPCR) (Jia-Sheng Xu et al., 2020), and small cell lung cancer (validated by immunoblotting and qPCR) (Moss et al., 2009). *SSTR2,* as a G protein-coupled cell surface receptor, can be activated by extracellular ligands, which leads to the inhibition of cell proliferation (Lechner et al., 2021). Precious studies demonstrated that *SSTR2* might serve as a molecular target in the diagnosis and treatment of thyroid cancer (Thakur et al., 2021), small intestinal neuroendocrine tumor (Elf et al., 2021), and neuroendocrine tumors (Si et al., 2021). *VIP* can provide protection from apoptosis in tumorigenesis (Sastry et al., 2017). OXTR and its ligand oxytocin (OXT) are present in the gastrointestinal system and involved in tumorigenesis (Ma et al., 2019). *IL1A* was involved in various immune responses, inflammatory processes, and hematopoiesis, which might be associated with colon tumorigenesis (Yoshikawa et al., 2017). To our knowledge, the relationship between *GRP* and tumorigenesis has not been reported.

Furthermore, we systematically explored the characteristics of the tumor immune microenvironment. The results revealed that the tumor-infiltrating resting NK cells or M0 Macrophages, *TP53* mutation rates, and *TMB* could be independent prognostic signatures for colon cancer. Additionally, we observed that the expression levels of checkpoint genes (*PD-L1, PD-1*, and *CTLA-4*) were higher in high-risk score patients, which may suggest that our immune-related risk score model was capable of providing support for immunotherapy. More importantly, the immune-related signature was also significantly associated with OS in patients with anti-PD-L1 treatment. We speculated that patients with a low-risk score might be more sensitive to ICI therapy based on the result of Supplementary Figures S5, S6.

In addition, we compared the performance of our constructed immune-related prognostic model with the published prognostic model of colon cancer based on the cohorts TCGA-COAD, GSE39582, and GSE17536, which is summarized in Supplementary Table S7. Our constructed prognostic model was relatively and effectively validated in more internal and external cohorts, including an immunotherapeutic cohort.

## Conclusion

In summary, a robust immune-related prognostic model was constructed, and the characteristics of the tumor immune microenvironment were explored, which may be helpful for the prognosis and immunotherapy of colon cancer patients.

## Data Availability

Publicly available datasets were analyzed in this study. These data can be found in the following: the expression profiles and corresponding clinical information of TCGA-COAD were downloaded from https://xenabrowser.net/datapages/. GSE39582 and GSE17536 were downloaded from the NCBI-GEO database (https://www.ncbi.nlm.nih.gov/geo/); and the immunotherapeutic cohort (IMvigor210) was available according to the guideline on http://research-pub.gene.com/IMvigor210CoreBiologies using the IMvigor210CoreBiologies R package.
