# Supplementary figures and images for: Exploring Immune-Related Prognostic Signatures in the Tumor Microenvironment of Colon Cancer
Source: Front Genet. 2022 Feb 24;13:801484. doi: 10.3389/fgene.2022.801484 (PMC8907673; doi:10.3389/fgene.2022.801484)

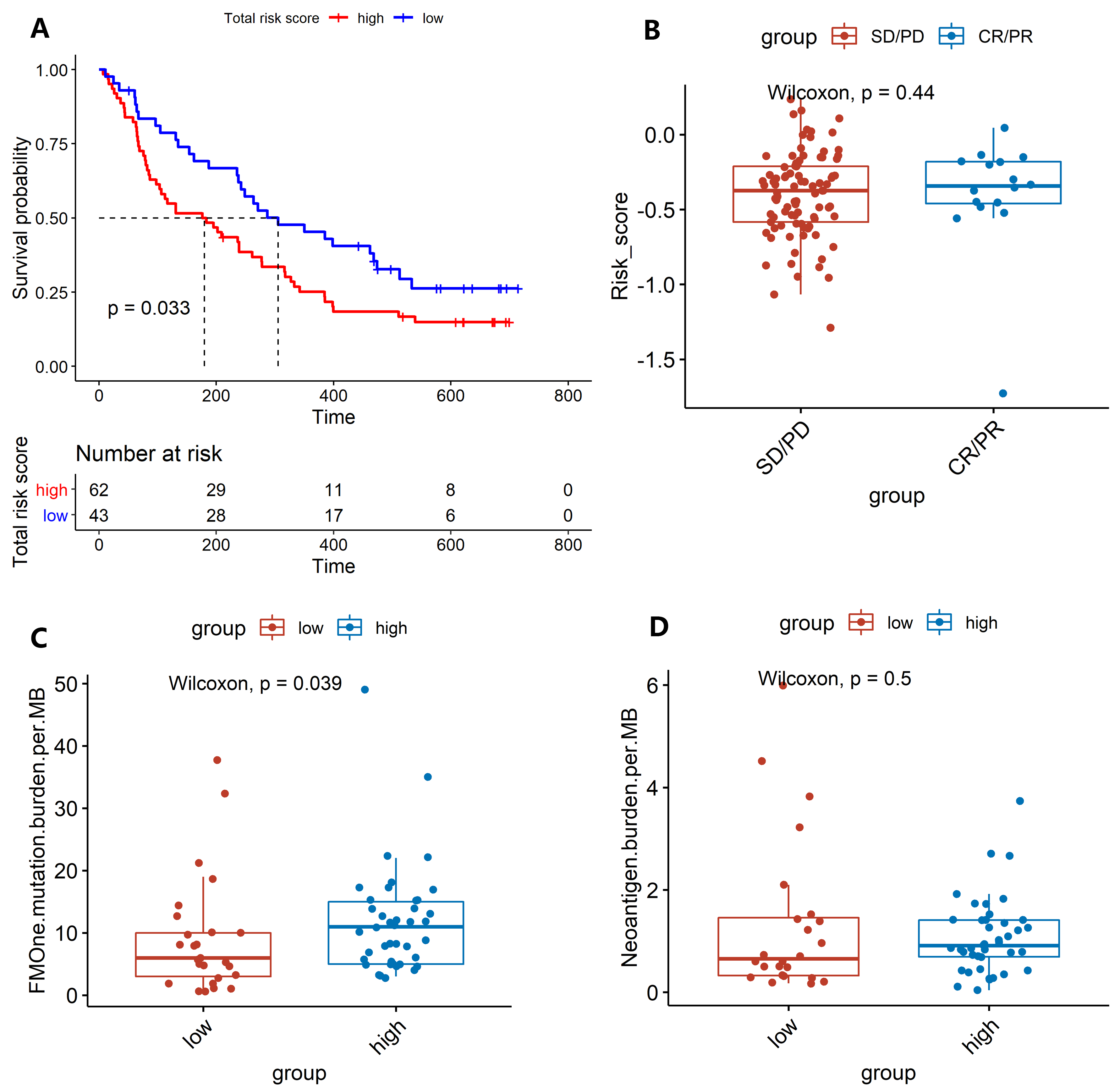

Supplement: Supplementary file 3 [file Image6.TIF]

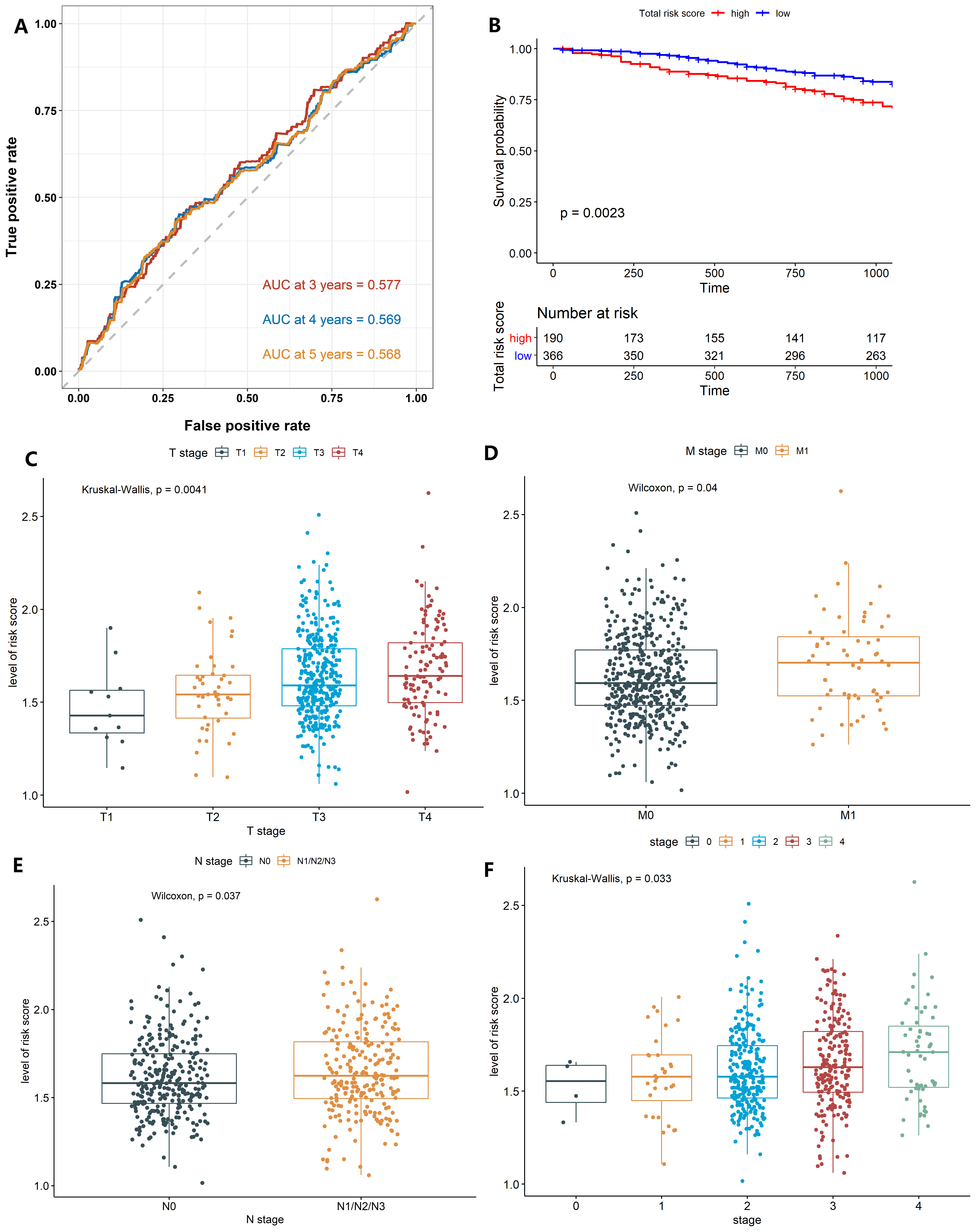

Supplement: Supplementary file 4 [file Image3.TIF]

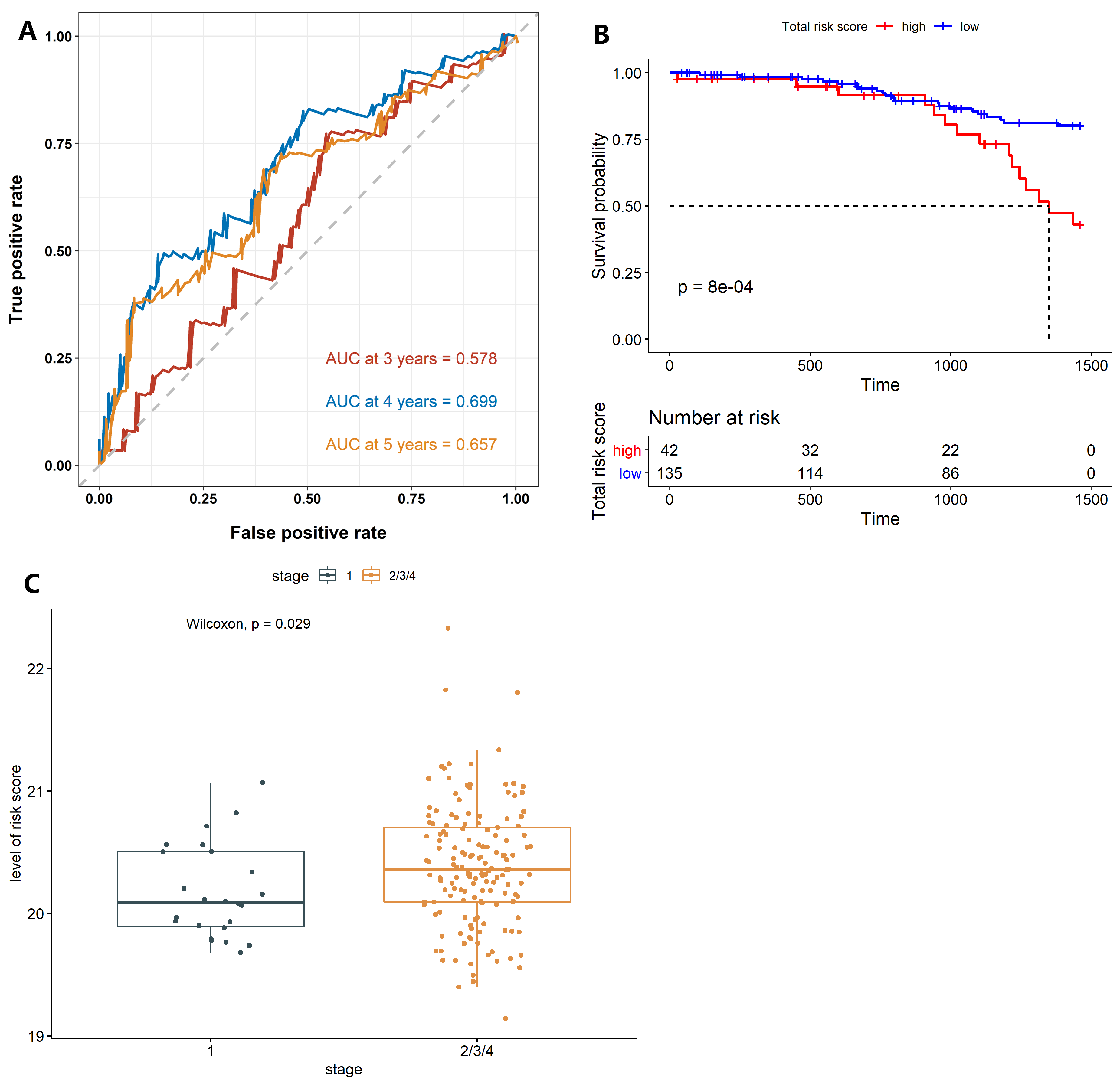

Supplement: Supplementary file 5 [file Image4.TIF]

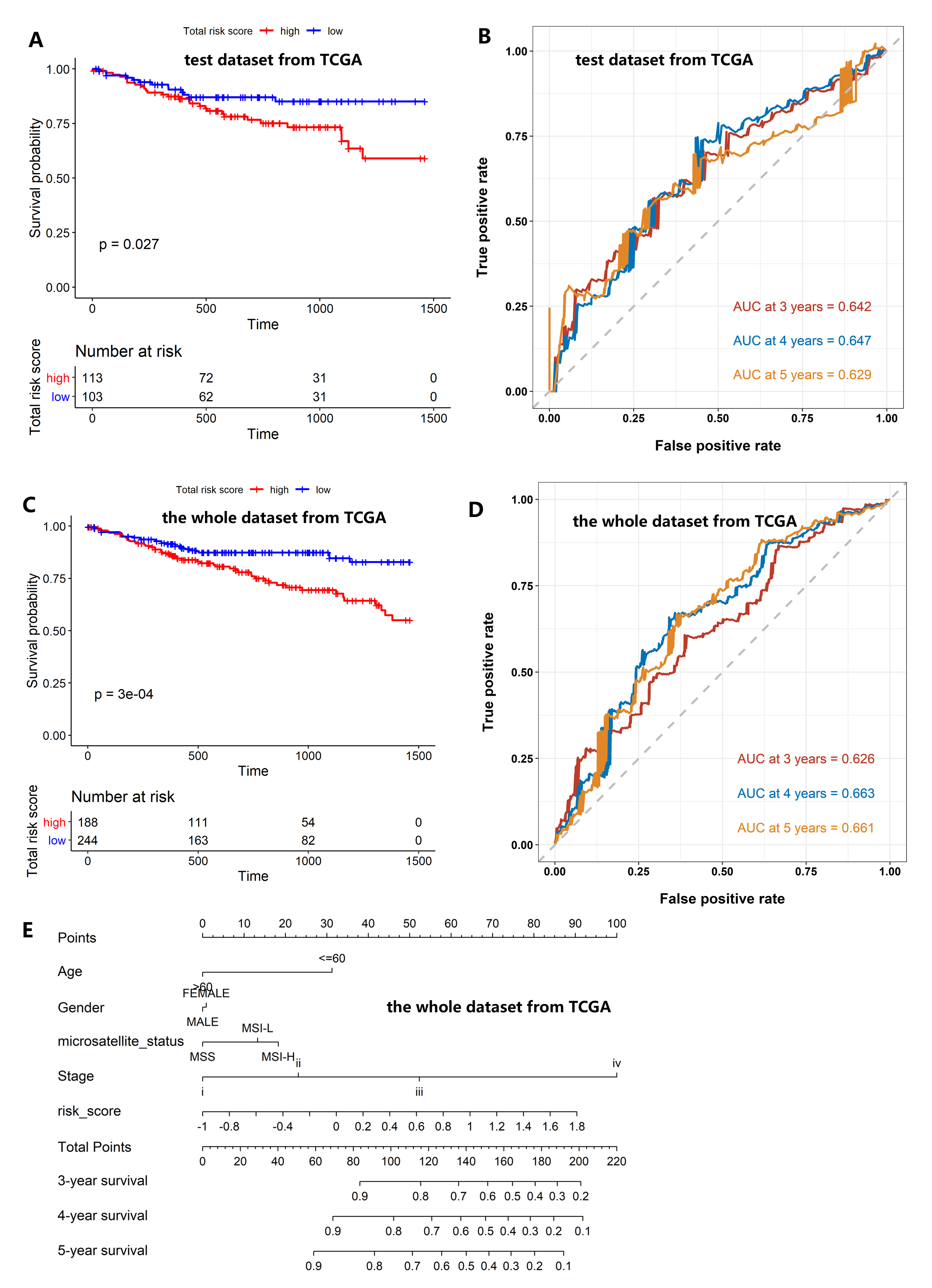

Supplement: Supplementary file 6 [file Image2.TIF]

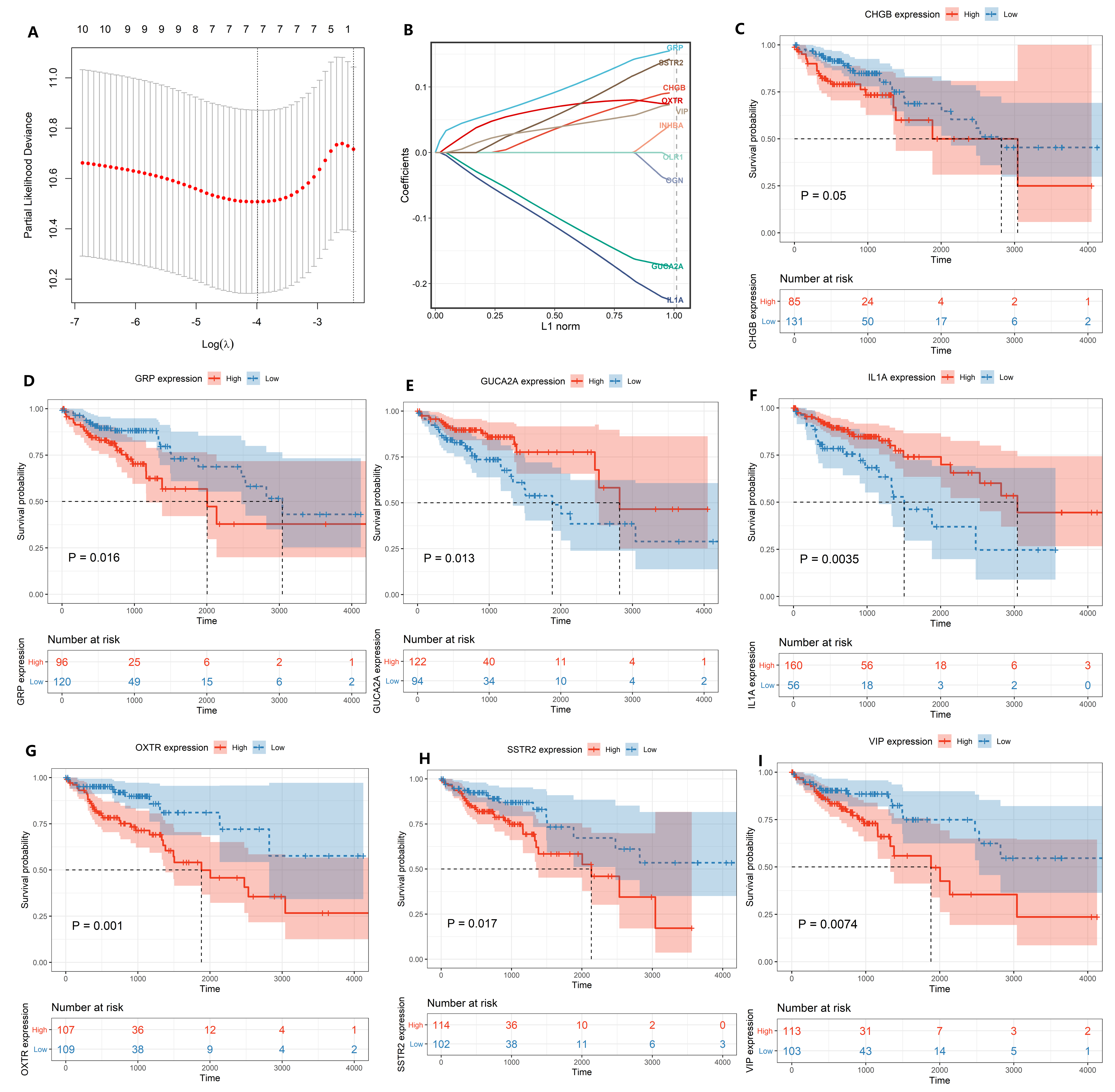

Supplement: Supplementary file 7 [file Image1.TIF]

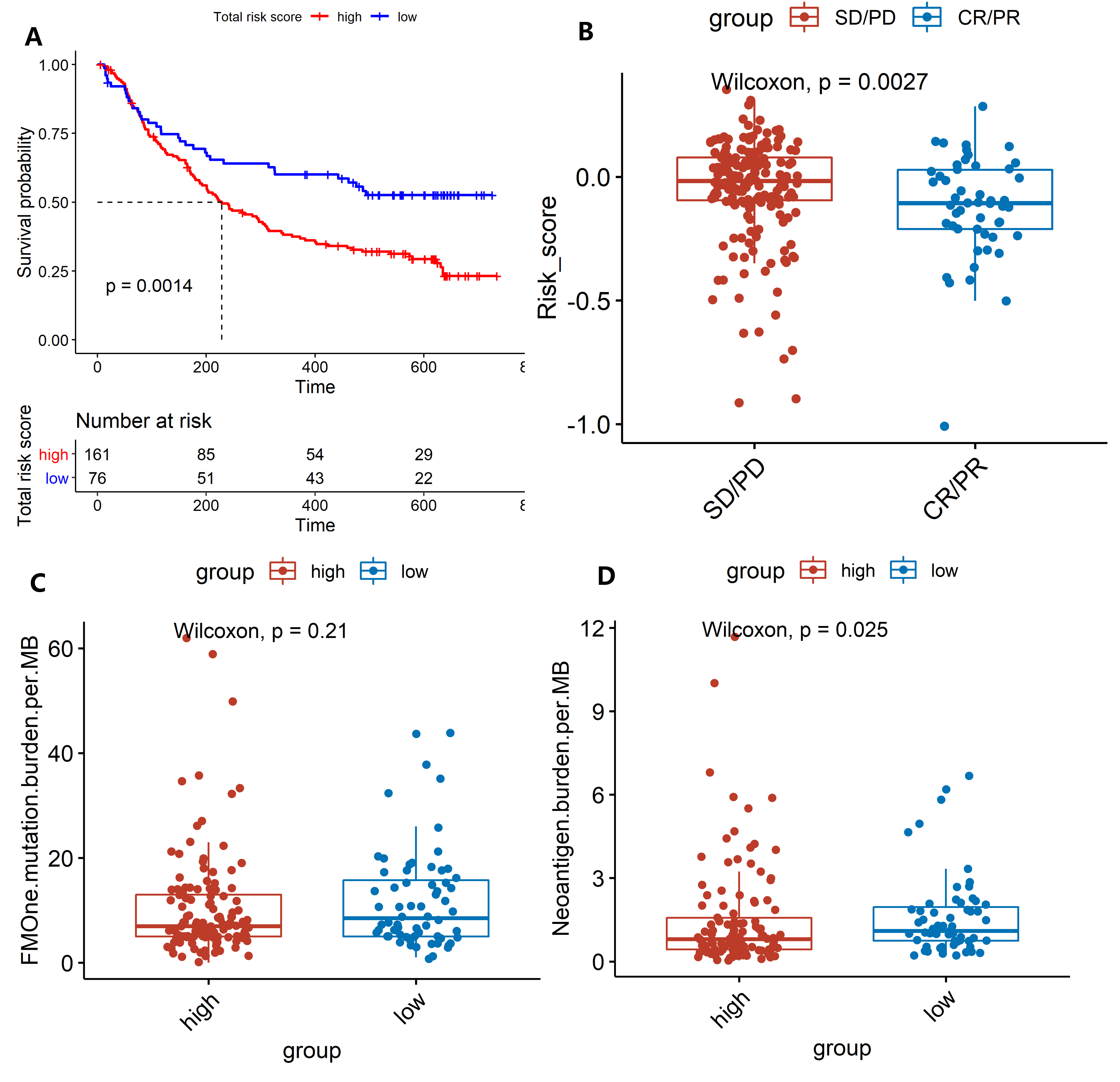

Supplement: Supplementary file 11 [file Image5.TIF]
